# Editorial: Case reports in pediatric gastroenterology, hepatology and nutrition 2022

**DOI:** 10.3389/fped.2023.1206993

**Published:** 2023-05-25

**Authors:** Benjamin Rakotoambinina, Jan Vagedes

**Affiliations:** ^1^ Lab LRI Division of Isotopic Medicine, Pediatric and Adult Physiology, University of Antananarivo, Antananarivo, Madagascar; ^2^ ARCIM Institute, Department of Pediatrics, Filderklinik, University of Tübingen, Filderstadt, Germany; ^3^ ARCIM Institute, Department of Neonatology, Filderklinik, University of Tübingen, Filderstadt, Germany

**Keywords:** pandysautonomia, distal esophageal spasm, peutz-Jeghers syndrome, fibrinogen disorders, caroli disease, torque teno virus related hepatitis, scurvy, pediatric digestive clinical case report

**Editorial on the Research Topic** Case reports in pediatric gastroenterology, hepatology and nutrition 2022

## Introduction

The combined fields of gastroenterology, hepatology, and nutrition form a holistically evolving foundation with segmented subspecialties linked around the gastrointestinal (GI) tract and its appendages. Another key driver is macro/micro nutrition, closely linked to interplaying with the gut-immune system and the GI microbiome extended to the gut-brain axis, these subfields benefit from the technical support of functional GI assessments, super-resolution multimodal imaging and child-sized diagnostic/interventional endoscopy (Int-Endosc). The dynamics of the field stimulate translational research, inspiring thriving publications, including Clinical Case Reports (CCRs).

While much of the scientific interest during the COVID-19 pandemic focused on the importance of randomized controlled trials (RCTs), large case series, and meta-analyses (1) addressing SARS, Cov-2 and vaccines, it is now time to refocus on other “thematic organ systems,” including underutilized scientific approaches such as CCRs (2). These typically have the potential to describe novel discoveries and cutting-edge treatment modalities, providing a valuable source of new concepts and suscitating questioning knowledge for clinical care in the broad field of pediatric gastroenterology.

Previously, CCRs in Frontiers in Pediatrics were scattered among different sections. In 2022, the Editorial Board initiated this Research Topic (RT) to focus explicitly on CCRs, to which we have brought our collective interest and expertise**.** The aim of this RT was to gather outstanding findings and advances in pediatric gastroenterology and related fields.

## Results

Five CCRs were accepted after a rigorous interactive blinded peer review process involving 75 experts from various countries.

Jing Sun et al. reported a case of Caroli's disease (CD), a rare congenital malformation with intrahepatic bile duct dilatation that presents a challenging diagnostic process. This 13-year-old boy presented with an unexplained and recurrent fever, non-specific abdominal pain, and episodic jaundice. He was started on an appropriate antibiotic regimen for suspected suppurative cholangitis. He underwent an extensive work-up, including magnetic resonance cholangiopancreatography (MRCP), which showed specific features of CD biliary ectasia. This relatively early diagnosis has avoided serious complications that can include extensive hepatic abscess, biliary infection, pancreatitis, and, in late stages, cholangiocarcinoma in the case of an erroneous or delayed diagnosis. This CCR outlines the relevance of MRCP, an imaging tool with highly discriminative diagnostic performance, to expedite the challenging characterization of malformed hepatic bile ducts. Its early application avoids a “diagnostic odyssey” and the poor outcome of a child with CD condemned to liver transplantation (3).

In mid-2022, Yujiao Zhou et al. reported a case of acute hepatitis in a 10-year-old-boy with no history of COVID-19. The boy presented with abdominal pain, hepatic splenomegaly (confirmed by ultrasound), and jaundice associated with high bilirubin levels and elevated transaminases (alanine-aminotransferase 2,330 U/L and aspartate-aminotransferase 1,326 U/L). MRCP showed edema of the periportal region and a stenotic choledochus. A comprehensive search for common etiologies was undertaken. This excluded standard viral infections (e.g., SARS COV-2, 41-adenovirus, Epstein-Barr, herpes simplex virus, and cytomegalovirus) and autoimmune hepatitis. The authors reached a diagnosis of non-AE hepatitis (WHO criteria). Next-generation sequencing, metagenomic NGS, and hepatic biopsy unexpectedly revealed Torque-Teno Virus (TTV) in the blood and liver. Plasmapheresis with a boost of corticosteroids improved hepatic function leading to his discharge. This CCR addressed the hot topic of mystery hepatitis by identifying, for the first time, TTV as a potential cause of this liver immune dysregulation during the omicron Covid-19 wave (4).

The case report by Kakiuchi et al. describes an interesting case of GI bleeding in a 7-year-old boy with Peutz-Jeghers syndrome (PJS) and hypofibrinogenemia following Int-Endosc mucosal resection of small intestinal polyps. Genetic characterization of this fibrinogen disorder revealed a heterozygous pathogenic mutation in exon 10 of the fibrinogen gamma chain. In this case, we see the unusual occurrence of two separate monogenic diseases in one individual. This CCR expands our understanding of conditions that may be associated with PJS and outlines key aspects of the diagnosis of PJS and congenital abnormalities of fibrinogen. The key learning point from this CCR was the importance of taking steps to prevent postoperative bleeding during polypectomy in individuals with PJS. Hypofibrinogenemia should be considered a risk factor for such bleeding (5).

Hanhua Zhang et al. describe the first case of a 12-year-old boy diagnosed simultaneously with two rare conditions: (i) acute pandysautonomia (A-PDA), a post-ganglionic sympathetic and parasympathetic failure, and (ii) distal esophageal spasm (DES), a motility disorder. Without any relevant family history, this boy presented with recurrent dysphagia, nausea, vomiting and autonomic instability (e.g., decreased lacrimation and sweating but over-salivation, pupillary dilation, and urinary retention).

After considering the differential diagnoses, A-PDA was confirmed by MR imaging, which showed enhancement of the lumbar nerve root in the left fiber endings. This case of A-PDA did not respond to treatment with intravenous immunoglobulin. A combination of oryzanol, mecobalamin, and lorazepam improved his vomiting. However, he still exhibited dysphagia and residual postprandial vomiting with growth retardation requiring nasogastric feeding. Upper GI endoscopy revealed a contraction of the lower esophagus with a contractile ring. Results of high-resolution esophageal manometry and barium swallowing confirmed the diagnosis of DES. His family refused plasmapheresis, even though it is recommended for such a condition. The selected treatment option was per oral endoscopic myotomy (POEM), a minimally invasive procedure that has gained more popularity in adults than in children. POEM resulted in a significant relief of the patient's symptoms, as exemplified by a reduction in the commonly used Ekcardt score from 9 to 2 postoperatively. This CCR highlights the first use of a cutting-edge technique (POEM) for DES in a child who was also diagnosed with A-PDA, another rare condition (6).

A 6-year-old boy with autism, acute malnutrition, and undiagnosed scurvy experienced a pulmonary arterial hypertension (PAH) crisis that resulted in cardiac arrest during endoscopic assessment, as reported by Quinn et al. The risk of experiencing anesthesia in a patient can be greatly increased by life-threatening cardiovascular events due to a hidden selective malnutrition. In addition, a severe but reversible form of PAH has been linked to vitamin C deficiency. The authors undertook a comprehensive review of the relevant literature, proposed a novel clinical algorithm for risk stratification and mitigation prior to anesthesia in patients at high risk for scurvy and associated PAH, and discussed how to take the next step toward better patient outcomes. This CCR raises awareness of the importance of considering pre-anesthesia micronutrient assessment in children (7).

## Concluding remarks

This RT focusing on digestive CCRs in Frontiers Pediatrics follows the trend of o the renowned journals to set aside a separate location for the collection of CCR (8, 9). This RT is noteworthy because it addresses various topics spanning the GI, hepatic, Int-Endosc, and nutritional fields, particularly a secular micronutrient deficiency. Each CCR is rich in key takeaways for readers and clinicians, which makes them so interesting.

Overall, CCRs are a useful way to expand knowledge about rare and unusual complications of common or orphan diseases. Furthermore, a re-evaluation of collections of CCRs may even bring to light valuable data with practical real-life implications (10). The CCR platform is also of great interest to busy gastroenterologists searching for a specific topic.

**Figure 1:**
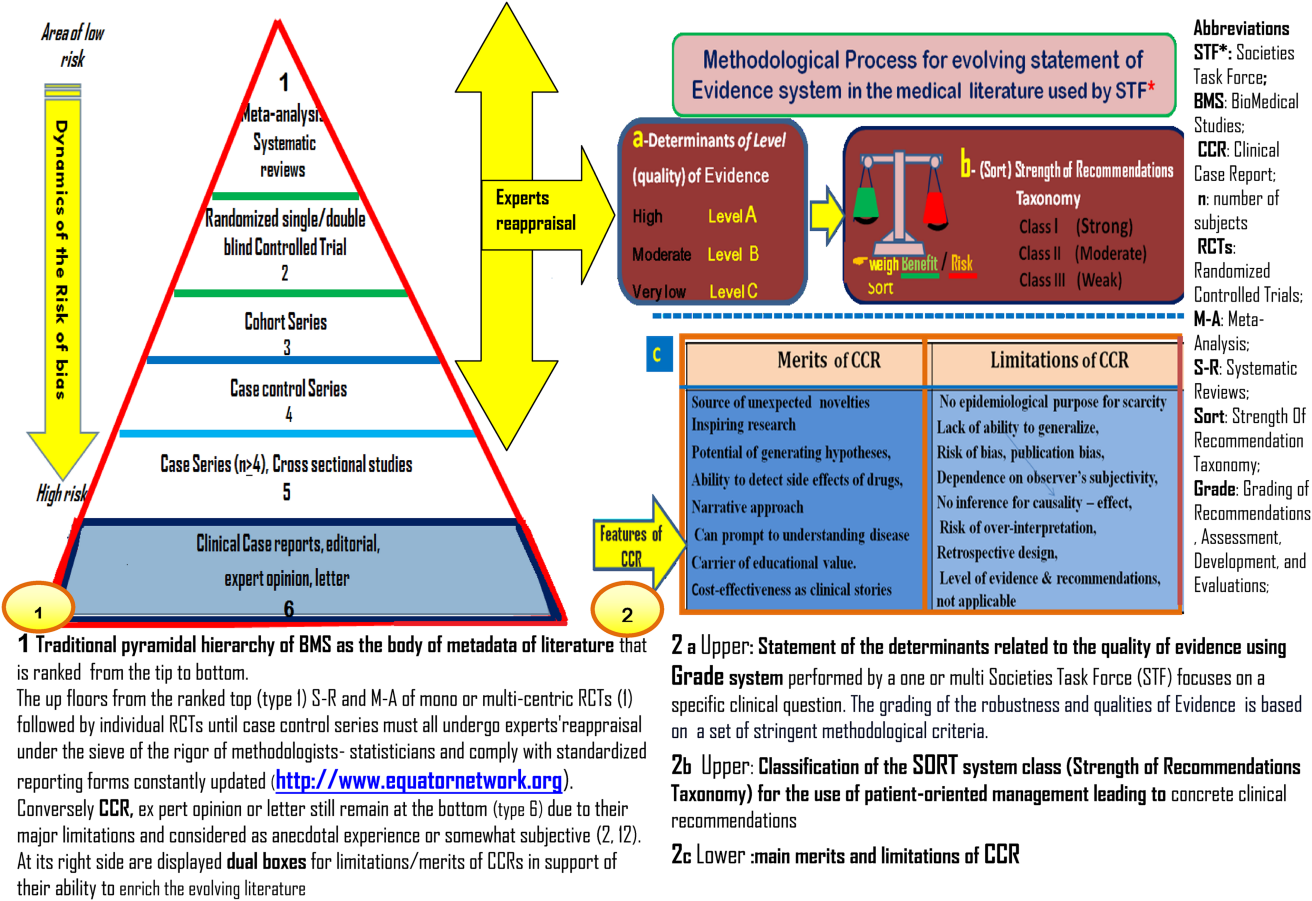
Schematic representation of biomedical studies for use in clinical practice guidelines and recommendations.

This RT hopefully serves to justify the publication of CCRs, an often-undervalued format at the bottom of the pyramid of scientific studies (Figure 1). The use of current methodological quality requirements should further enhance the value of CCRs, allowing them to continue to contribute to advances in clinical practice (11–14).
