# Supplementary material for: Editorial: Case reports in pediatric gastroenterology, hepatology and nutrition 2022
Source: Front Pediatr. 2023 May 25;11:1206993. doi: 10.3389/fped.2023.1206993 (PMC10249014; doi:10.3389/fped.2023.1206993)
Supplement: Supplementary file 1 [file Datasheet1.pdf]

Revised Supplementary material linked to Figure.1
Legend of Figure.1: Schematic representation of BioMedical Studies for use in clinical practice guidelines and recommendations)
Title Editorial : Case Reports in Pediatric Gastroenterology, Hepatology and Nutrition 2022

Topic Editors Frontiers
Benjamin Rakotoambinina[1], Jan Vagedes[2]

Legend:

1 **Traditional pyramidal hierarchy of BMS as the body of metadata of literature** that is ranked from the tip to bottom.

The up floors from the ranked top (type 1) S-R and M-A of mono or multi-centric RCTs (1) followed by individual RCTs until case control series must all undergo reappraisal under the sieve of the rigor of methodologists & statisticians and comply with standardized reporting forms such as PRISMA (for S-R and M-A), not limited to, that are constantly updated by the equator network (**http://www.equator-network.org**).

Conversely CCR, ex pert opinion or letter still remain at the bottom (type 6) due to their major limitations and considered as anecdotal experience or somewhat subjective (2, 12). At its right side are displayed **two boxes** for limitations and merits of CCRs in support of their ability to enrich the existing literature or serve as a starting point for reflective perspectives of further research.

2a Upper 2a**: Statement of the determinants related to the quality of evidence using Grade system**

This Process of Grading Evidence, usually performed by a one or multi Societies Task Force (STF) focuses on a specific clinical question. GRADE system permits to assess the study quality of the evidence according to impactful criteria such as risk of bias, indirectness, heterogeneity or inconsistency, imprecision, and publication bias. The robustness of evidence and qualities of evidence are graded as:

- **I**. High when STF grants firm confidence that the TCE is closed to the ECE
- **II.** with two sub-grades: **II**a. *Moderate* and **II**b**.** *Low*
- **III.** Very low when STF gives very limited credit to the study

2b Upper 2b: **Classification of the SORT system class (Strength of Recommendations Taxonomy) for the use of patient-oriented management**

It is the turning point moving towards concrete recommendations (SORT). The determinants of quality of the studies rely on the scrutiny of any limitation with the appraisal of balance of benefit versus risk and cost-efficiency. They are decreasingly graded as Class **A** (strong), **B** (moderate) and **C** (weak).

2c Lower 2c **: merits and limitations of CCR**

☞abbreviations. **STF*:** Societies Task Force**; BMS**: BioMedical Studies; **CCR**: Clinical Case Report; **n**: number of subjects in the studies; **RCTs**: Randomized Controlled Trials; **M-A**: Meta-Analysis; **S-R**: Systematic Reviews; **Sort**: Strength Of Recommendation Taxonomy; **Grade**: Grading of Recommendations, Assessment, Development, and Evaluations; **TCE**: True Clinical Effect; **ECE**, Estimated Clinical Effect.
